# Disability pension dynamics in early adulthood: A two-decade longitudinal study of educational, work and welfare-state trajectories in Norway

**DOI:** 10.1016/j.ssmph.2022.101062

**Published:** 2022-03-13

**Authors:** Sina Wittlund, Arnstein Mykletun, Thomas Lorentzen

**Affiliations:** a Nordland Hospital Trust, Regional Competence Centre for Work and Mental Health, PO Box 1480, 8092, Bodø, Norway; b Department of Community Medicine, UiT - The Arctic University of Norway, PO Box 6050 Langnes, N-9037, Tromsø, Norway; c Department of Sociology, University of Bergen, PO Box 7802, 5020, Bergen, Norway

**Keywords:** Disability pension, Mental disorders, Prevention, Social policy, Register data, Educational, work and welfare trajectories, WHO, World Health Organisation, OECD, Organisation for Economic Co-operation and Development, NAV, Norwegian Labour and Welfare Administration (orignially an Norwegian abbreviation of Nye arbeids- og velferdsetaten), NIPH, Norwegian Institute of Public Health, ICPC, International Classification of Primary Care, ICD, International Classification of Diseases, IA, Norwegian Inclusive Workplace Agreement (Norwegian abbreviation of Inkluderende arbeidsliv-avtalen), NOK, Norwegian Kroner

## Abstract

**Background:**

Since the 1990's, structural transformations in the Norwegian economy have decreased employment opportunities for low-skilled young people lacking formal education credentials. In parallel with these economic changes, there has been a strong increase in the proportion of young disability pensioners. Preventing labour market exit requires a thorough understanding of the disability process. We aim to 1) identify the most typical trajectories into disability pension for young Norwegian inhabitants between 1993 and 2014 and 2) investigate if the trajectories and composition of young disability pensioners changed over time.

**Methods:**

Using high-quality Norwegian registry data, we established two population-based cohorts of Norwegian inhabitants aged 29–39 years in either 2003 (cohort 1) or 2014 (cohort 2) who were not disability pensioners during the first month of their cohort period but had been granted a disability pension by the cohort end-date. Cohort 1 was followed from the beginning of 1993 through 2003, cohort 2 from 2004 through 2014. We used sequence and cluster analyses to identify typical disability pension trajectories and investigate how they changed overtime.

**Results:**

The majority follow trajectories characterised by little or no previous work participation. Both the trajectories and composition of young disability pensioners changed overtime. Between the two cohorts there was 1) a doubling in the probability of following 'precarious income trajectories', 2) a decrease in the probability of following ‘work and/or education trajectories’ and 3) an increase in the proportion of early school leavers.

**Conclusion:**

Current initiatives such as the Norwegian Inclusive Workplace Agreement (IA) focus on preventing transitions from employment to disability benefits. However, such initiatives have little relevance for young disability pensioners as the majority have weak labour market attachment. Policymakers should therefore consider placing more emphasis on non-workplace interventions.

## Highlights

• The majority of young Norwegian disability pensioners are early school leavers with weak labour market attachment.
• Disability benefits may increasingly cater for young people based on social (rather than medical) needs.
• Preventing disability pension dependency in early adulthood requires a thorough understanding of the disability process based on knowledge of typical trajectories.
• Sequence analysis is a novel approach within the medical sciences and has been used to identify holistic life course trajectories.

## 1 Introduction

### 1.1 Background

For decades there has been a slow, but steady increase in the proportion of young disability pensioners both in Norway (Brage & Thune, 2015) and in other OECD countries (OECD, 2013). In Norway more than 10.5% of working age adults receive disability benefits (Statistics Norway, 2021) which is the highest proportion of disability pension beneficiaries in the OECD (Hemmings & Prinz, 2020). In recent years there has been a sharp increase in the proportion of people aged 18–29 on disability benefits (Ellingsen, 2017). This is a significant economic burden for Norwegian society and a very poor outcome for the individual as the majority will be dependent on disability pension for up to 45 years (OECD, 2013).

### 1.2 Young disability pension and mental disorders

In Norway, more than 65 percent of young disability pensions are granted due to mental disorders (Ellingsen, 2017) which is a far greater proportion than for older age groups (OECD, 2013). Between 2010 and 2016 there was a sharp spike in disability pension incidence among young Norwegian inhabitants aged 18–29, primarily due to escalating numbers of 25-29-year-olds granted disability benefits due personality and behavioural disorders (Ellingsen, 2019). This has been accompanied simultaneously by a substantial increase in self-reported mental health ailments among Norwegian adolescents and young adults (NIPH, 2019), as well as significant rise in proportion of young people registered with an International Classification of Primary Care (ICPC-2-R) diagnostic code (WONCA, 2005) for mental symptoms from primary care (NIPH, 2019). However, currently there is not sufficient background data available to determine secular trends in the prevalence of mental disorders in Norway (NIPH, 2016).

### 1.3 Risk factors for young disability pension

Previous studies show that psychiatric diagnosis, non-completion of secondary education, low socioeconomic status and disadvantaged social background are strong independent predictors of young disability pension (De Ridder et al., 2013; Krokstad et al., 2002; Myhr et al., 2018). There is also evidence that immigrants from low-income countries, especially males from North Africa and the Middle East, have higher risks of disability pension receipt in young adulthood than ethnic Norwegians (Claussen et al., 2012). Intergenerational transmission of welfare is another disturbing phenomenon in modern welfare states. Dahl et al. (2014) find that when parents are granted disability pension, the likelihood that one of their adult offspring also becomes dependent on disability benefits rises significantly over the next decade.

## 2 Contextual background for the study

### 2.1 Societal context

Since the early 1990's, structural transformations in the Norwegian economy have led to a decline in labour market participation for adults with low educational attainment. First, there was a tremendous expansion in tertiary education accompanied simultaneously by labour market automation due to new information and communication technologies (ICTs), including innovations such as machine learning and digitalisation of production (Frey & Osborne, 2013; Nedelkoska & Quintini, 2018). This created a knowledge-intensive, skill-biased labour market where technological advances favoured groups with higher educational attainment and reduced the employability of other groups, particularly low-wage earners, working in unskilled positions (Hansen & Lorentzen, 2019). Their risk of job displacement was higher, and the number of workforce positions requiring only low qualification decreased dramatically until as few as 5% of available jobs required no formal education (OECD, 2010). Second, unskilled Norwegian workers with a low level of education were largely displaced by workers from Central and Eastern Europe who immigrated to Norway in 2006-09 (Bratsberg et al., 2014; Jean & Jimenez, 2007).

### 2.2 The Norwegian labour and welfare administration

Prior to 2006 the labour and welfare administration in Norway was based on three different public service institutions (Employment Services, Social Insurance Administration, and Municipal Social Services) with limited collaboration between institutions. From 2006 to 2011, the Norwegian Labour and Welfare Administration (NAV) reform was implemented by merging these three institutions into a “one-stop-centre” called NAV that is responsible for all employment and welfare services in Norway. A paramount objective of the reform was to improve labour market integration for vulnerable groups by providing access to a greater range of rehabilitation services and job training programmes (Dahl & Lorentzen, 2017).

Norwegian disability pensions are administered by NAV and can be granted to inhabitants with at least 50% reduced earning capacity due to illness or injury (NAV, 2019a). Disability benefits compensate 66 percent of the recipients average income, up to a salary cap of 6 times the National insurance basic amount (G) (NAV, 2019a). If a recipient has low or no previous income, they are entitled to a minimum benefit between NOK 242 590 - 309 621, which varies depending on the recipient's age and relationship status (NAV, 2019a; NAV, 2020). Outflow from disability pension to self-supporting employment is negligible, Norway has one of the lowest disability benefit claim rejection rates and there are no systematic assessments once a disability benefit has been granted (Hemmings & Prinz, 2020). Disability pension is therefore considered a permanent state within the Norwegian benefit system.

A particularly relevant innovation of the aforementioned NAV reform was the introduction of a generous new benefit, the Work Assessment Allowance (WAA) in 2010. Compared to its predecessors, WAA has relatively liberal qualification criteria and provides individuals without prior labour force participation access to the full range of NAVs work-oriented measures. Previously, these benefits were only available to people with labour-market experience.

To be entitled to WAA, an individual's work capacity must be reduced by at least 50%, primarily due to illness or injury (Folketrygdloven, 2017). NAV evaluates the individual's health and functionality in addition to their ability to meet work performance requirements of normal income-generating employment (NAV, 2019b). This work capacity assessment is based on information provided by the applicant. Eligibility for WAA also requires a diagnosis. Significantly however, ICPC-2 symptom diagnosis can be approved if a formal International Classification of Diseases (ICD) diagnosis (WHO, 2016; WHO, 2019) has not been established.

Physicians who are both members of Norwegian Social Insurance Medical Association^1^ as well as expert advisors to NAV are concerned that eligibility criteria for WAA may increase medicalisation of young people's social needs (Ministry of Labour and Social Affairs, 2016). They report that the utilisation of symptom diagnoses, coupled with the absence of non-medical based benefit alternatives, results in many individuals (especially young people) being granted a health-related benefit (WAA) even though the primary cause of their reduced function is social problems (Ministry of Labour and Social Affairs, 2016). Physician concerns regarding medicalisation of young people's social problems are backed-up by recent research from both Norwegian economists (Markussen & Røed, 2020) and social scientists (Bakken, 2020).

WAA was expected to improve the rates of young people re-entering the workforce. However, it has not reaped the intended benefits as the possibility of transforming this temporary entitlement into a permanent disability pension after several years undermines the seriousness of vocational integration efforts (OECD, 2013). In fact, critics have coined WAA “a waiting ground for disability pension” (Kann & Kristoffersen, 2014).

### 2.3 The Norwegian Inclusive Workplace Agreement

In 2001, The Norwegian Inclusive Workplace Agreement (IA-avtalen) was initiated through collaboration between the government, business organisations and labour unions (The Norwegian Government, 2018; Ministry of Labour and Social Affairs, 2021). An overarching goal of the IA is to prevent transitions from work life to disability benefits through initiatives aimed at reducing sickness absence and improving work environments (Ministry of Labour and Social Affairs, 2021). However, without in-depth knowledge of typical trajectories into disability pension, we cannot know if such initiatives were appropriate for our study population.

### 2.4 Aims and expectations

Developing policies and interventions to prevent young disability pension requires a thorough understanding of the disability process based on knowledge of common trajectories. We therefore aim to 1) identify the most typical educational, work and welfare-state trajectories into disability pension for two cohorts of young Norwegian inhabitants between 1993 and 2014 and 2) investigate if the trajectories and composition of young disability pensioners changed overtime.

Given large-scale economic and institutional transformations that took place during the study period, we expect to see changes in the background composition of the two study cohorts. We anticipate that there will be a greater proportion of early school leavers in the later cohort as there are simply fewer opportunities for them in the modern Norwegian labour market. For the same reasons we predict that young people in the second cohort are more likely to follow trajectories characterised by a precarious income situation with low or no previous labour market experience.

We also expect to see inter-cohort differences related to changes in social welfare policy. Our assumption is that the disability process takes longer for the latest cohort as a result of a longer period spent in health-related rehabilitation after the introduction of WAA in 2010. In regard to gender, we expect women to be overrepresented in both cohorts as there is evidence that women have a higher likelihood of disability pension than their male counterparts (Haukenes et al., 2012).

## 3 Methods

### 3.1 Data source

For this study, we used administrative data collected by Statistics Norway. The dataset contained longitudinal and detailed individual information on demography, education, income, work, and social welfare benefits for the complete Norwegian population starting in the early 1990s. The extensive information allowed the longitudinal reconstruction of life courses over a long-time span. In the original data most time-varying variables were recorded with exact start and stop dates, although some variables, such as those collected from tax-registers and educational registers were recorded annually. The quality and consistency of the administrative records used for the analyses is in general very high and undergo strict quality control from Statistics Norway before being made available for research purposes.

### 3.2 Analytical sample and cohorts

The study population was Norwegian inhabitants aged 29–39 years in either 2003 (cohort 1) or 2014 (cohort 2) who were not disability pensioners during the first month of their cohort period but had been granted a disability pension sometime between the second and last month of the respective follow-up periods. Cohort 1 was followed from the beginning of 1993 through 2003, cohort 2 from 2004 through 2014. The age restriction provided us with a homogenous population with regards to life-phase stages, such as schooling, labour market experience, and family phase. In total this gave us a population of 19 300 from cohort 1 and 15 964 from cohort 2.

### 3.3 Study design

We used Sequence analysis (SA) to identify typical trajectories leading into disability pension. SA has been used extensively over the last few years within the social sciences to identify holistic life course trajectories, but less so within the medical sciences. Utilising SA allows the study of how transitions are interconnected and constitute complex life courses, while at the same time reducing some of the complexity and heterogeneity found over the full range of individual sequences. This can be seen as a contrast to the focus on single transitions found in more traditional regression-based approaches (Aisenbrey & Fasang, 2010). Our analytical approach followed a three-step procedure. Each step was performed separately for the two cohorts.

The first step involved the calculation of distances between sequences (Gabadinho & Ritschard, 2013). In more practical terms, the distance is the result of the number of changes that need to be done to make two sequences similar (Brzinsky-Fay, 2007). The more similar they are, the less is the distance between them. The distance calculation is based on two types of changes, insertions/deletions (indels), and substitutions. Substitution costs were here user defined and derived from state attributes using the Gower distance (Studer & Ritschard, 2014). The Gower dissimilarity coefficient was based on the qualitatively assessed distance from work for each of the states, as well as information of whether the status type was a job, a health-related benefit, other welfare state benefits, or education. This resulted in a measure ranging from 0 to 1, where 1 is the maximum defined distance between states. Following common procedure, the indels cost were defined at 0.5, which is half the maximum cost of substitutions.

The second step followed the clustering procedure to identify typical trajectory types into disability. The clusters were identified using a clustering approach, where hierarchical clustering (Ward) was used as starting values for partitioning around medoids (PAM) clustering (Studer, 2013). Clustering quality was assessed using a range of cluster quality measures found in the Weighted cluster package in R (Studer, 2013). The best cluster solutions produced 6 distinct trajectory types for the 2003 cohort, and 7 for the 2014 cohort.

In the last step, we ran multinomial logistic regressions on the relationship between explanatory variables (presented below), and the trajectory types identified in the clustering procedure. The regression framework allowed us to consider compositional differences within and between cohorts. For ease of presentation, the regression analyses were presented as average marginal effects, thereby avoiding well known methodological problems when comparing logits or odds ratios (Breen et al., 2018; Mood, 2010).

## 4 Variables and measures

### 4.1 Statuses

Nine mutually exclusive monthly statuses covering school, work, and social welfare were defined (Table 1). In the instance of overlapping states, such as, e.g., part-time work and disability, the highest placed status in the status alphabet (Table 1) determines the overall monthly status. The system of preference was based on the conception that more permanent and/or disadvantaged states overrules states that are less permanent and/or disadvantaged.

**Table 1:** Monthly statuses.

Status	Description	
Disability pension	Registered with disability pension current month	● Destination state for the whole study-population ● Presupposes at least 50% reduced work capacity ● Considered a permanent state within the Norwegian benefit system.
Health-related rehabilitation	Registered with either: temporary disability benefit, vocational rehabilitation benefit or medical rehabilitation benefit current month (prior to 2010), or the work assessment allowance current month (from 2010)	● Collective term used for uptake of one of four temporary health-related rehabilitation benefits. ● The three health-related rehabilitation benefits that were avalaible prior to 2010 (temporary disability benefit, vocational rehabilitation benefit, medical rehabilitation benefit) were merged into one category for the statistical-analysis. ● The aim of collapsing these three benefits into one category was to avoid unnecessary complexity as well as to achieve comparability across cohorts and over the full observation period.
Social assistance	Registered with means tested social assistance benefits current month	● A means tested benefit which is considered to be the last safety net in the Norwegian social welfare system. ● Considered meagre from both from a Norwegian and an international perspective (Lorentzen & Dahl, 2020).
Sickness benefit	Registered with sickness allowance current month	● Regarded as extremely generous by compensating sickness at a 100% of current income. ● Only available to those who have earned the right through work. ● Maximum duration of one year ● Possibility to transfer to less generoushealth-related rehabilitation benefits after one year.
Unemployed O.H.	Registered as unemployed and occupational handicapped/reduced working capacity current month	● Status given to unemployed people waiting for rehabilitation ● Assessed by NAV as having reduced working capacity.
Unemployed	Registered as ordinary unemployed current month	● Status assigned to those who have registered as ordinary unemployed at their local NAV office. ● The category includes both those with earned rights to unemployment benefit and those without.
Education	Registered under education current month if month is in a year with a valid educational record and none of the above statuses apply current month	● Status assigned if educational activity was registered the current month. ● In cases where education was combined with statuses in the social security system, the latter was given preference.
Work	Registered with a spell of work current month	● Status designated to those registered as participating normal, income-generating employment.
Other	If none of the above statuses apply	● Status containing those with unknown alternative income sources who were not registered as employed, in education or receiving any welfare benefits.

### 4.2 Explanatory variables

In the regression analyses, we entered several demographic and socioeconomic variables motivated by previous research on the transition into disability. Country background was categorised into three broad groups consisting of Norway, which served as reference category, Western Europe/North America/Oceania, and Non-western countries. For the gender variable, men served as a reference group. Furthermore, due to the instrumental importance of education, we have separated between those who have finished upper secondary education and those who have not. Persons who had finished upper secondary education by the age of 30 served as reference category.

Socioeconomic background was analysed using parental education and parental disability pension. Parental education was measured for the parent with the longest education in years using the Norwegian Standard Classification of Education (NUS2000) normalised from 0 to 1 for the presentation of average marginal effects. Thus, for the interpretation of the effect of parental education, a one-unit-change is interpreted as the distance between the lowest and the highest parental education observed. Parental disability benefit dependency is measured for both parents when I/O were 29 years old. No parents on disability pension serves at the reference category for the multivariate analyses. A dummy-variable indicating whether one lived in an urban or a rural community at t0 served as a proxy for the prevailing labour market conditions.^2^

## 5 Results

In Table 2, we present and compare status durations in months. We find that the disability pension process changed over time. People in the first cohort spent more time on disability pension, 57.6 months in cohort 1 vs 43.5 months in cohort 2 (t < 0.01) (Table 2), which signifies that the disability pension process took longer in the later cohort. While the duration of health-related rehabilitation is more or less the same for both cohorts, the average number of months spent unemployed increased considerably in the later cohort. This is solely due to a 12% (t < 0.01) increase in the use of the unemployment category “occupational handicapped” (Table 2). The average duration of work participation decreased from 10.2 months in cohort 1 to 5.8 months in cohort 2 (t < 0.01) (Table 2), while the average number of months where young people were supported economically through alternative income sources (alternative maintenance) increased 67.9% (t < 0.01) (Table 2).

**Table 2:** Cohort-specific status duration in months.

Cohort-specific status duration in months:	2003-cohort	2014-cohort	Sig.a
Disability pension	57.6	43.5	**
Health-related benefits	31.3	32.6	**
Social assistance	8.7	9.6	**
Sickness allowance	6.5	5.3	**
Unemployed, occupational handicapped	1.8	15.0	**
Unemployed, ordinary	5.5	5.8	**
Education	3.0	3.5	**
Work	10.2	5.8	**
Other	7.4	10.9	**
Total (N)	132 (19 300)	132 (15 964)	

a Two-sample t-tests on differences in means between cohorts. *t < 0.05 **t < 0.01.

Over the observation period, the proportion of early school leavers increased from 50.1% to 62.3% (t < 0.01) (Table 3) and fewer people in the later cohort combined disability pension with some form of work, 26.1% in cohort 1 vs 20.3% in cohort 2 (t < 0.01) (Table 3). Women are overrepresented in both cohorts, although the gender gap decreased by 1.6% (t < 0.01) between cohorts (Table 3). The proportion of disability pensioners with a country background outside Norway increased 6.1% (t < 0.01) between cohorts (Table 3), mainly due to a 4.3% (t < 0.01) increase in disability pensioners from Non-western countries. In both cohorts approximately 50% of the study population had at least one parent who was also a disability pension recipient (Table 3).

**Table 3:** Cohort-specific descriptive statistics.

Cohort-specific descriptive statistics (%)	2003-cohort	2014-cohort	Sig.
Turbulence (mean)^c^	10.8	12.0	**a
Country background			**b
Norway	86.6	80.5	
Western Europe, North-America, Oceania	7.0	8.8	
Non-Western	6.4	10.7	
Gender			**b
Male	46.5	48.1	
Female	53.5	51.9	
Education			**b
Finished upper secondary education	49.9	37.7	
Early school leaver	50.1	62.3	
Parental education NUS level (mean)	3.0	3.3	
Region			**b
Urban	76.3	77.9	
Rural	23.7	22.1	
Parental disability status			**b
No parental disability pension	50.3	52.0	
One parent disabled	36.8	33.9	
Two parents disabled	12.9	14.1	
Parental ISEI (mean)^d^	39.6	NA	a
Work activity last month of observation			
No work activity	73.9	79.7	**b
Work 1–19 h a week	17.8	15.9	
Work 20–29 h a week	2.3	1.9	
Work 30+ hours a week	6.0	2.4	

a Two-sample t-tests on differences in means between cohorts. *t < 0.05 **t < 0.01.

b Pearson chi-square test on cohorts and non-metric variables. *t < 0.05 **t < 0.01.

c Turbulence is a composite measure reflecting the number of distinctive sequences and the time in each state (Elzinga & Liefbroer, 2007). Higher turbulence implied shorter spells and more shifts between statuses.

d The ISEI-scale translates occupational income and education into a continuous prestige-scale ranging from 10 to 90 (Ganzeboom, 2010; Ganzeboom et al., 1992).

### 5.1 Trajectories

**Fig. 1:**
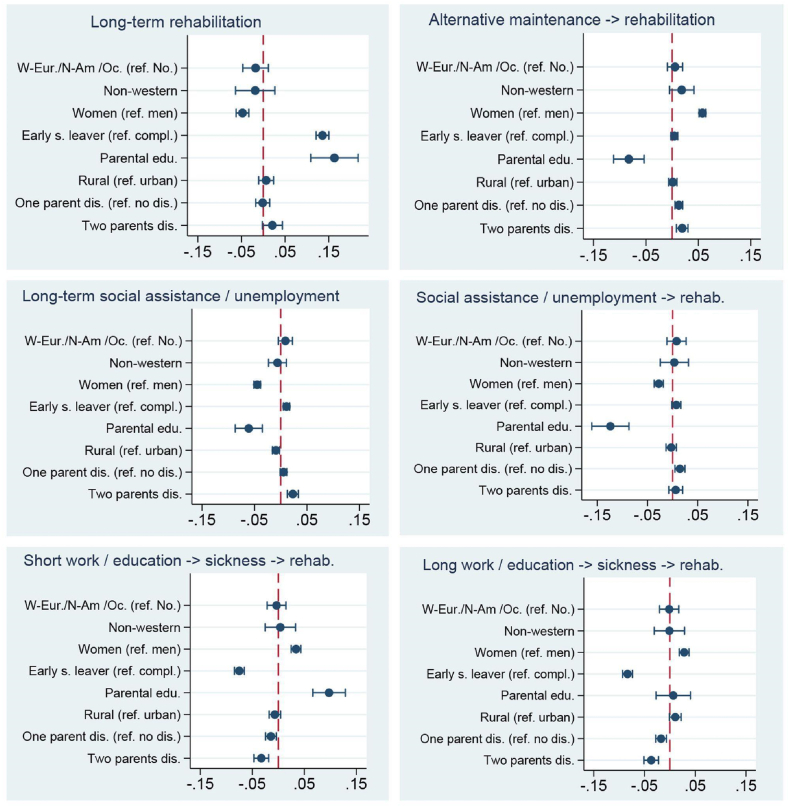
Cohort 1: Average marginal effects (AME).

**Fig. 2:**
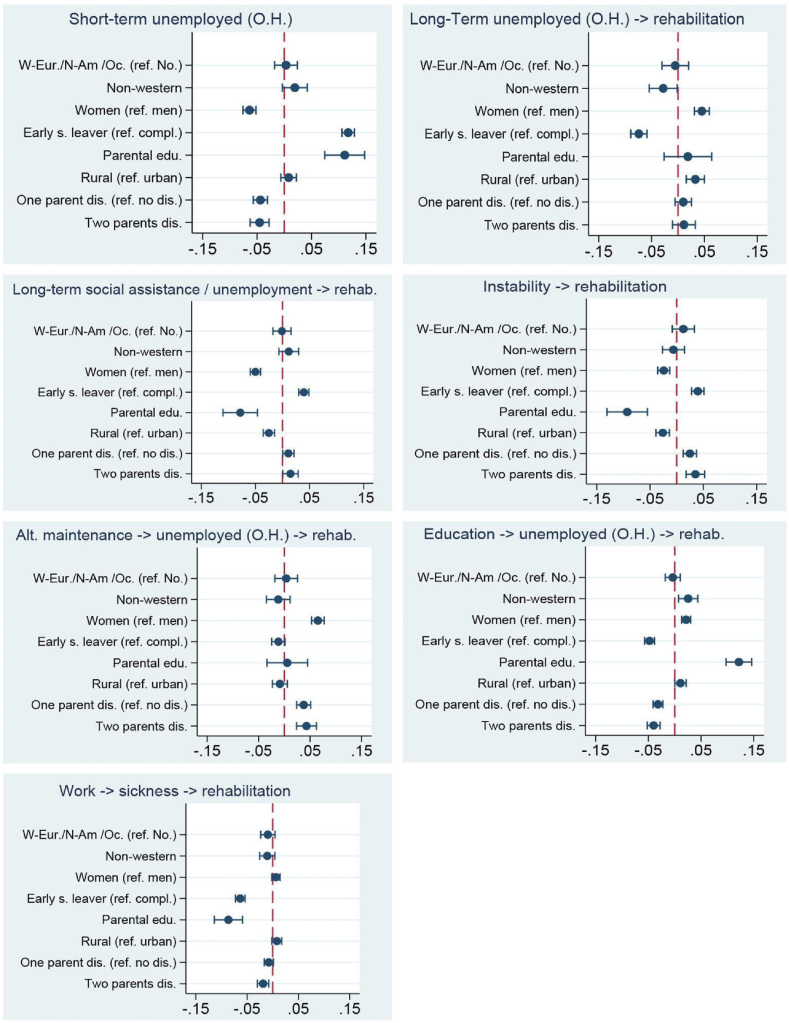
Cohort 2: Average marginal effects.

Next, we present the most typical trajectory types resulting from the optimal matching and clustering procedures along with the trajectory-specific risk factors depicted with average marginal effects (AME). The description of trajectories is based on a combination of information sequence index plots (Supplementary Appendix Figures A1, A2), plots depicting mean time in each state by cluster (Supplementary Appendix Figures A3, A4) and plots depicting average marginal effects (Fig. 1, Fig. 2). For those interested in obtaining an in-depth understanding of the trajectory types and their followers, we have included a set of cluster-specific background variables in Appendix Table 1.

Optimal matching and PAM clustering identified six distinct clusters for cohort 1 (Figure A1, Table 4) and seven distinct clusters for cohort 2 (Figure A2, Table 4). To simplify cohort comparison, trajectory types have been distributed into three broad categories, characterised as ‘Trajectories via work and/or education’, ‘Health related benefits trajectories’ and 'Precarious income trajectories'. Trajectory types and their defining characteristics are summarised in Table 4.

**Table 4:** Trajectory types and characteristics.

Type	Trajectories			
	2003-cohort	%	2014-cohort	%
Via work and/or education	(C5) Short work/education - > sickness - > rehab.: Characterised by a short spell of work/education leading into a period of sickness benefits followed by rehabilitation. After seven years, most followers have transitioned into disability pension.	11.3	(C6) Education - > unemployed (O.H) - > rehab.: Characterised by education at baseline followed by unemployment registered as occupational handicapped leading into rehabilitation followed by disability pension.	7.6
	(C6) Long work/education - > sickness - > rehab.: Long period of work and/or education leading into a period of sickness benefits followed by rehabilitation. After ten years, most followers have made the transition into disability pension.	12.1	(C7) Work - > sickness - > rehabilitation: Here we find persons who participated in stable competitive employment before entering sickness benefits followed by rehabilitation and disability pension.	6.7
Health-related benefits trajectories'	(C1) Long term rehabilitation: A typical sequence is characterised by regular unemployment leading into unemployment registered as occupational handicapped. This is followed by a long and uninterrupted period of health-related rehabilitation finally leading into disability pension.	54.1	(C1) Short-term unemployed (O.H): This trajectory is characterised by short-term, occupational handicapped unemployment transiting into disability pension after a very brief rehabilitation spell.	17.0
	-		(C2) Long-term unemployed (O.H.): The most typical sequence here is a long spell of occupational handicapped unemployment, then an abrupt transit into four years of health-related rehabilitation leading into disability pension.	26.0
Precarious income trajectories	(C2) Alternative maintenance - > rehabilitation: The most typical sequence starts with a long spell of alternative maintenance leading into rehabilitation followed by disability pension.	6.8	(C3) Long-term socass./unemp. - > rehab.: This trajectory is characterised by intervals of unemployment or social assistance leading into rehabilitation followed by disability pension.	10.0
	(C3) Long term social assistance/unemployment: This trajectory type is characterised by long-term labour market exclusion leading to disability pension.	5.0	(C4) Instability - > rehabilitation: This is a heterogenous trajectory consisting of general instability. Individuals following this trajectory type experienced frequent shifts between different states leading into rehabilitation and disability pension.	14.3
	(C4) Social assistance/unemployment - > rehab.: A typical sequence for this trajectory type is labour market exclusion leading into a short spell of rehabilitation followed by social assistance.	11.0	(C5) Alt. maintenance - > unemp. (O.H.) - > rehab.: The most typical sequence here is health-related rehabilitation (including O.H.) leading into an extended period of alternative maintenance followed by another period of health-related rehabilitation (including O.H.) before transferring to disability pension.	18.6

## 6 Cohorts

### 6.1 Cohort 1 (1993-2003) trajectories

#### 6.1.1 Trajectories via work and/or education (23.4%)

##### 6.1.1.1 Short work/education → sickness → rehabilitation (C5) - 11.3%

This cluster includes predominantly subjects with sequences that included a short-medium spell of labour market participation (Table 4). The average marginal effects (AME) depicted in Fig. 1 show that the trajectory probability increased with upper-secondary school completion, being female or having parents with a relatively high level of education. Parental disability pension decreased the chance of following this trajectory.

##### 6.1.1.2 Long work/education → sickness → rehabilitation (C6) - 12.1%

Here we find those who had a relatively long spell (6–7 years) of labour market participation prior to disability pension (Table 4). Regression analysis (Fig. 1) found that the trajectory-probability increased with upper secondary school completion and being female. Again, parental disability pension decreased the risk for this trajectory type.

#### 6.1.2 Health-related rehabilitation trajectories (54.1%)

##### 6.1.2.1 Long term rehabilitation (C1) - 54.1%

This trajectory represents the most common path to disability pension for cohort 1 (Table 4) and is characterised by a very high proportion of early school leavers. On average, being an early school-leaver increased the trajectory probability by 13.5% (Fig. 1). Being male and having parents with a relatively high level of education also somewhat increased the risk of following this trajectory (Fig. 1).

#### 6.1.3 Precarious income trajectories (22.5%)

##### 6.1.3.1 Alternative maintenance → rehabilitation (C2) - 6.8%

The heterogeneous ‘Other’ category is prominent here (Table 4). On average, women had a 5.8% higher chance of following this trajectory compared to men (Fig. 1). Other trajectory predictors were parental disability pension and having parents with a low-level of educational attainment.

##### 6.1.3.2 Long term social assistance/unemployment (C3) – 5%

This trajectory consists of long-term labour market exclusion leading to disability pension (Table 4). Being male or an early school leaver increased the trajectory probability by 4.5% and 1.1% respectively (Fig. 1). In addition, parental disability pension, low parental level of education and urbanicity inferred slightly increased risk.

##### 6.1.3.3 Social assistance/unemployment −> rehabilitation (C4) - 11%

The typical sequence here is: labour market exclusion → short spell of health-related rehabilitation → disability pension. Regression analysis found that men had a 2.7% higher risk of following this trajectory compared with women (Fig. 1). Other risk factors were parental disability pension or having parents with a low level of educational attainment (Fig. 1).

### 6.2 Cohort 2 (2004-2014) trajectories

In cohort 2, a striking phenomenon occurs after 7 years when the majority abruptly transfer into health-related rehabilitation (Figure A2). This corresponds with the introduction of the Work Assessment Allowance (WAA) in March 2010. WAA is very dominant in six of the seven trajectories (Figure A2).

#### 6.2.1 Trajectories via workVia work and/or education (14.3%)

##### 6.2.1.1 Education −> unemployed (O.H) −> rehabilitation (C6) - 7.6%

This trajectory is composed of subjects who were studying at baseline. AMEs depicted in Fig. 2 show that the strongest trajectory predictors were upper secondary school completion and having parents with a relatively high level of education. Being female, a non-western immigrant or a rural dweller were also trajectory risk factors. Parental disability pension decreased the trajectory probability.

##### 6.2.1.2 Work −> sickness −> rehabilitation (C7) - 6.7%

Individuals following this trajectory participated in stable competitive employment prior to disability pension (Table 4). Those who completed upper secondary school had, on average, a 6.3% greater chance of following this trajectory compared to early school leavers (Fig. 2). Interestingly, having parents with a relatively low level of educational attainment inferred increased risk for this trajectory type while having two parents on disability benefits inferred slightly decreased risk.

#### 6.2.2 Health-related rehabilitation trajectories (42.9%)

##### 6.2.2.1 Short−term unemployed (O.H.) (C1) - 17%

Here we find those who transited into disability pension after a short spell of occupational handicapped unemployment (Table 4). The trajectory probability was higher for early school leavers (11.7%) and men (6.4%) (Fig. 2). Having parents with a relatively high level of education was also a predictive factor. The risk of following this trajectory decreased with parental disability pension.

##### 6.2.2.2 Long−term unemployed (O.H.) −> rehabilitation (C2) - 26%

The dominant feature here is a long spell of occupational handicapped unemployment (Table 4). Predictors of this trajectory type were almost the exact opposite of the previous trajectory (Fig. 2). Thus, upper secondary school completion or being female substantially increased the trajectory probability. Ethnic Norwegians and rural dwellers also faced a somewhat elevated risk of following this trajectory.

#### 6.2.3 Precarious income trajectories (42.9%)

##### 6.2.3.1 Long−term social assistance/unemployment → rehabilitation (C3) - 10.0%

This trajectory is characterised by intervals of unemployment or social assistance (Table 4). Being male or an early school leaver were important trajectory predictors (Fig. 2). Additional risk factors were parental disability pension, having parents with a relatively low level of educational attainment and urbanicity.

##### 6.2.3.2 Instability −> rehabilitation (C4) - 14.3%

Here we have a heterogenous trajectory consisting of general instability (Table 4). AME's (Fig. 2) show that the trajectory-probability was greater for men and early school-leavers. Likewise, low parental education, parental disability and urbanicity inferred an elevated risk.

##### 6.2.3.3 Alternative maintenance → unemployed (O.H.) → rehabilitation (C5) - 18.6%

This trajectory is distinguished by a combination of the ‘occupational unemployment’ category and the heterogeneous ‘Other’ category (Table 4). According to the AME analysis (Fig. 2) women had, on average, a 6.5% higher chance of following this trajectory compared to men. Having one parent with a disability pension was associated with 3.8% increased risk while having two parents receiving disability benefits elevated the risk slightly further to 4.3%.

## 7 Discussion

Developing policies and interventions to prevent young disability pension requires a thorough understanding of the disability process based on knowledge of common trajectories. In line with this, our study has two main aims: 1) identify the most typical educational, work and welfare-state trajectories into disability pension for two cohorts of young Norwegian inhabitants between 1993 and 2014 and 2) investigate if the trajectories and composition of young disability pensioners changed overtime.

### 7.1 Aim 1

In both cohorts, the majority of young disability pensioners are early school leavers, following trajectories characterised by little or no previous labour force participation. Current initiatives, such as the Norwegian Inclusive Workplace Agreement (IA), are primarily focused on preventing transitions from employment to disability pension. However, workplace prevention initiatives will have little impact on young disability pensioners as the bulk of this population have weak labour market attachment.

### 7.2 Aim 2

Differences in status duration between the two cohorts suggests that disability pension serves a slightly different clientele for the latest cohort. The average duration of work participation decreased 57% between cohort 1 and cohort 2, which implies different background characteristics as well as a different pathway into disability pension. Cohort-specific descriptive statistics support this by showing that the share of early school leavers was higher for the latest cohort, with high school dropout increasing by 12% between cohort 1 and cohort 2. Lack of work experience combined with lower completion rates of upper secondary education indicates that young people in the second cohort were less equipped to deal with the demands of a knowledge-intensive labour market compared to their predecessors. Both findings could reflect that formal education is increasingly important and might have changed the functional requirements for the labour market (OECD, 2010). Our findings build on previous research indicating that higher education may be protective against disability pension in the Norwegian context (Østby, Ørstavik, Knudsen, Reichborn-Kjennerud, & Mykletun, 2011).

This general decline in the ability to meet job performance requirements of normal income-generating employment may also explain why fewer people in the later cohort combined disability pension with some form of work. Polvinen et al., (2018) provide further support for our results. Firstly, they present evidence that disability pensioners with higher education are more likely to partake in some form of part-time work compared to those with low education attainment. Secondly, they find that previous labour market attachment is associated with working after disability retirement (Polvinen et al., 2018). In addition, their results indicate that disability pensioners due to mental disorders are less likely to work than disability pensioners due to other conditions (Polvinen et al., 2018).

The overall increase in the proportion of early school leavers; the near doubling in the share following “precarious income trajectories”; as well as the concurrent decrease in the probability of following “work and education trajectories”, indicates that the function of disability pension may be changing. In the later cohort, it appears that disability pensions cater for people with primarily social (rather than medical) needs to a greater extent than the first cohort. Our observation is reinforced by previous Norwegian research demonstrating that disability benefits may function as an economic safety net for individuals with low education attainment who struggle with employment (Østby, Ørstavik, Knudsen, Reichborn-Kjennerud, & Mykletun, 2011).

There are concerns that the WAA (a health-related benefit introduced in 2010), increases medicalisation of young people's labour market struggles (Ministry of Labour and Social Affairs, 2016; Bakken, 2020; Hansen & Lorentzen, 2019). Eligibility for WAA requires a diagnosis; however, ICPC-2 symptom diagnosis can be approved if a formal ICD diagnosis has not been established. Utilisation of symptom diagnoses, coupled with the absence of non-medical based benefit alternatives, may result in young people being granted a health-related benefit (WAA) even though their reduced function can primarily be attributed to social factors (Ministry of Labour and Social Affairs, 2016). From WAA, the majority do not enter the labour market as intended but rather transition to permanent disability benefits (Kann & Kristoffersen, 2014). It is, however, beyond the scope of our paper to explore if medicalisation contributed to the sharp increase in young disability pensioners due to mental disorders after WAA was introduced.

### 7.3 Other important findings

Our analysis also provides evidence that gender plays a role in low-skilled young people's transitions from school to disability pension. In both cohorts, young men are more likely to follow trajectories characterised by labour market exclusion while women have higher probability of following alternative maintenance trajectories or trajectories via work and/or education. Women are overrepresented in both cohorts, although the gender gap decreased slightly over the study period. Cohort 2 is more ethnically diverse than cohort 1, which is likely due to the influx of adult immigrants to Norway from Non-western countries over the study's observation period (Jakobsen & Lorentzen, 2019).

Finally, our findings support previous research demonstrating that parental disability pension is associated with both low educational attainment and disability in their offspring (Dahl et al., 2014; Myhr et al., 2018). In both cohorts, approximately 50% of young disability pensioners had at least one parent who was also a disability pension beneficiary. Furthermore, low parental educational achievement is identified as a significant risk factor for following precarious income trajectories. Even though our research does not provide causal evidence, family vulnerability appears to be a key element in explaining this association.

### 7.4 Strengths

Using high-quality population-level registry data enables us to avoid typical problems associated with longitudinal surveys such as low response rates and high dropout rates. Furthermore, in contrast with more conventional methods such as cross-sectional and event history analyses, we are able to investigate how transitions are interconnected and how trajectories develop over time through complex, extended life courses.

### 7.5 Limitations

Future studies should include detailed health information so that one can assess how different diagnostic groups influence young people's educational, work and welfare-state trajectories into disability pension. We plan to repeat the analyses on data updated to 2020 and include detailed health information. Another limitation concerns extrapolation. The context of this study makes it primarily relevant for countries with extensive welfare states and knowledge-intensive labour markets. Finally, our analysis does not provide causal evidence. We have identified some important developments that should be further scrutinised by means of causal identification strategies.

## 8 Conclusion

### 8.1 Practical implications

Investigating young disability pension trajectories using sequence analysis has provided us with some valuable new insights. The majority of our study population are early school leavers with little or no previous labour market attachment. As such, workplace prevention strategies, such as the Norwegian Inclusive Worklife Agreement (IA) would have had limited impact on this group. Policymakers should therefore consider placing more emphasis on non-workplace interventions.

### 8.2 Future research

Given the strong intergenerational correlation in disability pension dependency, further research on causal mechanisms underlying intergenerational transmission of welfare could help prevent future disability pension within at-risk families. In addition, considering the sharp increase in young disability pensioners due to mental disorders, it would be interesting to investigate how different mental disorders influence young people's transitions from school to disability pension. It would also be interesting to investigate the role medicalisation may play in increasing the number of young Norwegian inhabitants on permanent disability benefits.

## CRediT authorship contribution statement

**Sina Wittlund:** Conceptualization, Study design, Methodology, Formal analysis, Literature Review, Visualization, Writing – original draft, Writing – review & editing. **Arnstein Mykletun:** Supervision, Conceptualization, Study design, Funding acquisition, Resources, Writing – review & editing. **Thomas Lorentzen:** Supervision, Conceptualization, Study design, Methodology, Formal analysis, Project administration, Resources, Software, Validation, Visualization, Writing – review & editing.

## Declaration of competing interest

None.

## Footnotes

1 The Norwegian Social Insurance Medical Association (Ntmf) is a specialist association within the Norwegian Medical Association.

2 Based on Statistics Norway's classification standard.
