# Supplementary material for: Disability pension dynamics in early adulthood: A two-decade longitudinal study of educational, work and welfare-state trajectories in Norway
Source: SSM Popul Health. 2022 Mar 13;17:101062. doi: 10.1016/j.ssmph.2022.101062 (PMC8933578; doi:10.1016/j.ssmph.2022.101062)

**Appendix**

Table A1 Trajectory Descriptives, cohort 1

| **Cluster-specific descriptive statistics, Coh. 1:** | **Cluster 1** | **Cluster 2** | **Cluster 3** | **Cluster 4** | **Cluster 5** | **Cluster 6** | **Total (N)** |
| --- | --- | --- | --- | --- | --- | --- | --- |
| Turbulence (mean) | 7,28 | 13,95 | 19,35 | 14,95 | 12,15 | 15,89 | 10,75 (19300) |
| *Country background* |  |  |  |  |  |  |  |
| Norway | 88,97 | 74,02 | 80,65 | 84,64 | 87,5 | 86,38 | 86,59 (16712) |
| Western Europe, North-America, Oceania | 6,77 | 8,71 | 6,94 | 6,88 | 7,37 | 7,09 | 7,03 (1357) |
| Non-Western | 4,26 | 17,27 | 12,41 | 8,47 | 5,13 | 6,53 | 6,38 (1231) |
| Year of birth (mean) | 1969,28 | 1968,75 | 1969,02 | 1968,99 | 1969,05 | 1968,44 | 1969,07 (19300) |
| *Gender* |  |  |  |  |  |  |  |
| Male | 48,49 | 24,62 | 71,82 | 54,55 | 38,28 | 39,95 | 46,47 (8969) |
| Female | 51,51 | 75,38 | 28,18 | 45,45 | 61,72 | 60,05 | 53,53 (10331) |
| *Education* |  |  |  |  |  |  |  |
| Finished upper secondary education | 43,00 | 53,33 | 45,46 | 48,36 | 66,11 | 66,72 | 49,89 (9457) |
| Early school leaver | 57,00 | 46,67 | 54,54 | 51,64 | 33,89 | 33,28 | 50,11(9498) |
| Parental education NUS level (mean) | 2,99 | 2,73 | 2,73 | 2,77 | 3,18 | 3,04 | 2,97 (18427) |
| *Region* |  |  |  |  |  |  |  |
| Urban | 75,86 | 76,89 | 79,71 | 76,28 | 78,14 | 75,11 | 76,32 (14090) |
| Rural | 24,14 | 23,11 | 20,29 | 23,72 | 21,86 | 24,89 | 23,68 (4372) |
| *Parental disability status* |  |  |  |  |  |  |  |
| No parental disability pension | 49,23 | 48,56 | 47,21 | 46,32 | 55,68 | 55,93 | 50,31 (9710) |
| One parent disabled | 37,27 | 36,59 | 35,02 | 40,2 | 34,52 | 34,45 | 36,78(7098) |
| Two parents disabled | 13,5 | 14,85 | 17,77 | 13,48 | 9,8 | 9,62 | 12,91 (2492) |
| Parental ISEI (mean) | N/A | N/A | N/A | N/A | N/A | N/A | N/A |
| *Work activity last month of observation* |  |  |  |  |  |  |  |
| No work activity | 72,14 | 89,17 | 91,9 | 83,25 | 66,9 | 63,83 | 73,88 (14258) |
| Work 1-19 hours a week | 18,5 | 8,18 | 4,21 | 11,75 | 24,04 | 25,26 | 17,80 (3436) |
| Work 20-29 hours a week | 2,4 | 0,83 | 0,74 | 1,16 | 3,39 | 3,65 | 2,34 (452) |
| Work 30+ hours a week | 6,96 | 1,82 | 3,15 | 3,85 | 5,68 | 7,26 | 5,98 (1154) |

Table A2 Trajectory Descriptives, cohort 2

| **Cluster-specific descriptive statistics, Coh. 1:** | **Cluster 1** | **Cluster 2** | **Cluster 3** | **Cluster 4** | **Cluster 5** | **Cluster 6** | **Cluster 7** | **Total (N)** |
| --- | --- | --- | --- | --- | --- | --- | --- | --- |
| Turbulence (mean) | 4,76 | 12,45 | 16,3 | 14,61 | 12,23 | 13,03 | 14,94 | 12,03 (15964) |
| *Country background* |  |  |  |  |  |  |  |  |
| Norway | 80,31 | 82,81 | 76,35 | 80,6 | 78,67 | 81,19 | 82,62 | 80,53 (12856) |
| Western Europe, North-America, Oceania | 9,11 | 8,59 | 8,18 | 9,06 | 9,35 | 8,62 | 7,71 | 8,79 (1403) |
| Non-Western | 10,57 | 8,59 | 15,47 | 10,33 | 11,98 | 10,19 | 9,67 | 10,68 (1705) |
| Year of birth (mean) | 1981,9 | 1980,06 | 1980,6 | 1980,21 | 1979,71 | 1982,76 | 1979,23 | 1980,53 (15964) |
| *Gender* |  |  |  |  |  |  |  |  |
| Male | 59,54 | 43,05 | 61,57 | 52,98 | 39,25 | 40,27 | 42,94 | 48,15 (7686) |
| Female | 40,46 | 56,95 | 38,43 | 47,02 | 60,75 | 59,73 | 57,06 | 51,85 (8278) |
| *Education* |  |  |  |  |  |  |  |  |
| Finished upper secondary education | 28,79 | 44,00 | 28,05 | 29,48 | 41,37 | 53,90 | 57,89 | 37,66 (5959) |
| Early school leaver | 79,21 | 56,00 | 71,95 | 70,52 | 58,63 | 46,10 | 42,11 | 62,34 (9865) |
| Parental education NUS level (mean) | 3,38 | 3,28 | 3,06 | 3,09 | 3,24 | 3,78 | 3,11 | 3,27 (15314) |
| *Region* |  |  |  |  |  |  |  |  |
| Urban | 77,34 | 75,46 | 81,97 | 80,75 | 78,83 | 76,64 | 75,07 | 77,86 (12227) |
| Rural | 22,66 | 24,54 | 18,03 | 19,25 | 21,17 | 23,36 | 24,93 | 22,14 (3477) |
| *Parental disability status* |  |  |  |  |  |  |  |  |
| No parental disability pension | 58,49 | 50,66 | 49,25 | 45,8 | 47,69 | 64,95 | 55,2 | 51,97 (8296) |
| One parent disabled | 29,21 | 35,03 | 34,91 | 37,13 | 36,82 | 26,51 | 33,36 | 33,92 (5415) |
| Two parents disabled | 12,3 | 14,32 | 15,85 | 17,08 | 15,49 | 8,53 | 11,43 | 14,11 (2253) |
| Parental ISEI (mean) | 39,19 | 39,5 | 36,9 | 37,49 | 39,92 | 44,11 | 37,87 | 39,23 (4919) |
| *Work activity last month of observation* |  |  |  |  |  |  |  |  |
| No work activity | 72,89 | 74,74 | 90,13 | 86,3 | 88,15 | 78,79 | 64,59 | 79,73 (12728) |
| Work 1-19 hours a week | 21,45 | 19,99 | 8,11 | 11,3 | 8,94 | 16,9 | 26,12 | 15,94 (2544) |
| Work 20-29 hours a week | 1,95 | 2,59 | 0,82 | 1,05 | 1,25 | 2,32 | 3,72 | 1,89 (302) |
| Work 30+ hours a week | 3,71 | 2,68 | 0,94 | 1,36 | 1,65 | 1,99 | 5,58 | 2,44 (390) |

Figure A1. Sequence index plot Cohort 1, sorted by mds


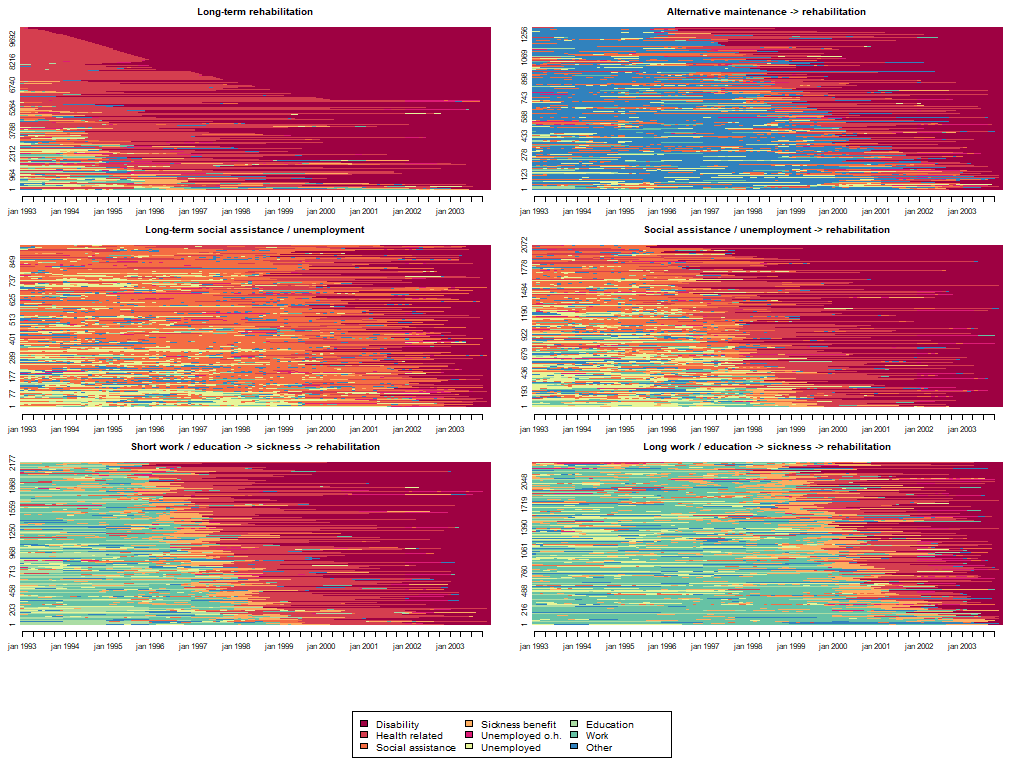


Figure A2. Sequence index plot Cohort 2, sorted by mds


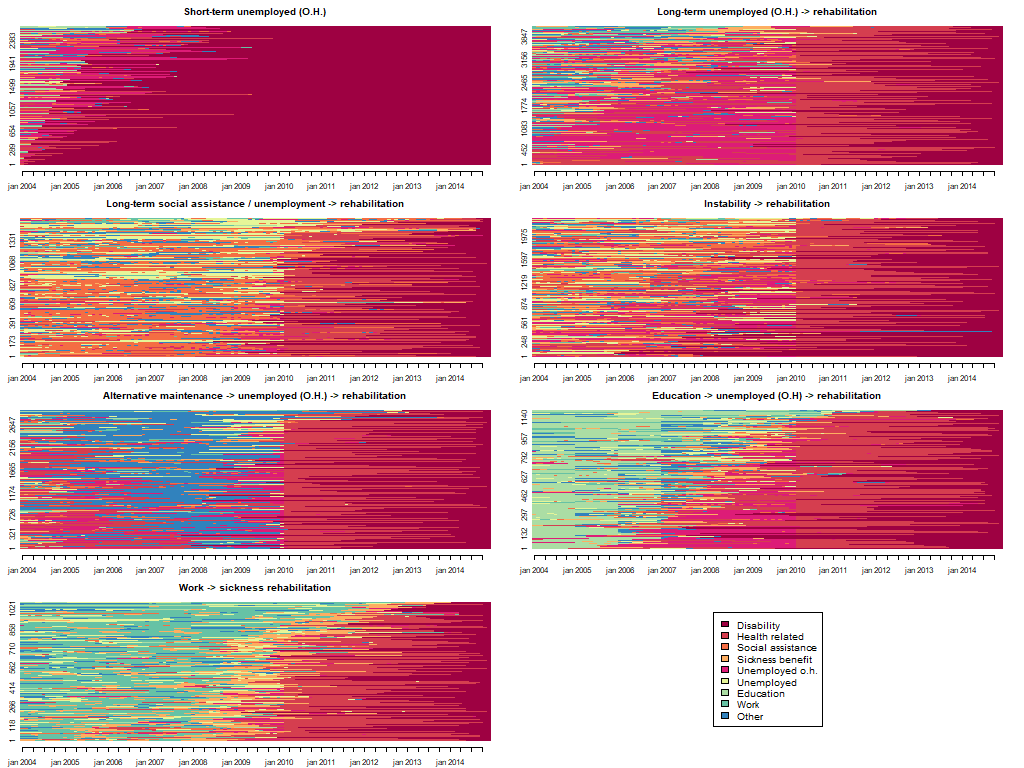


Figure A3. Mean time in each state for Cohort 1, by cluster


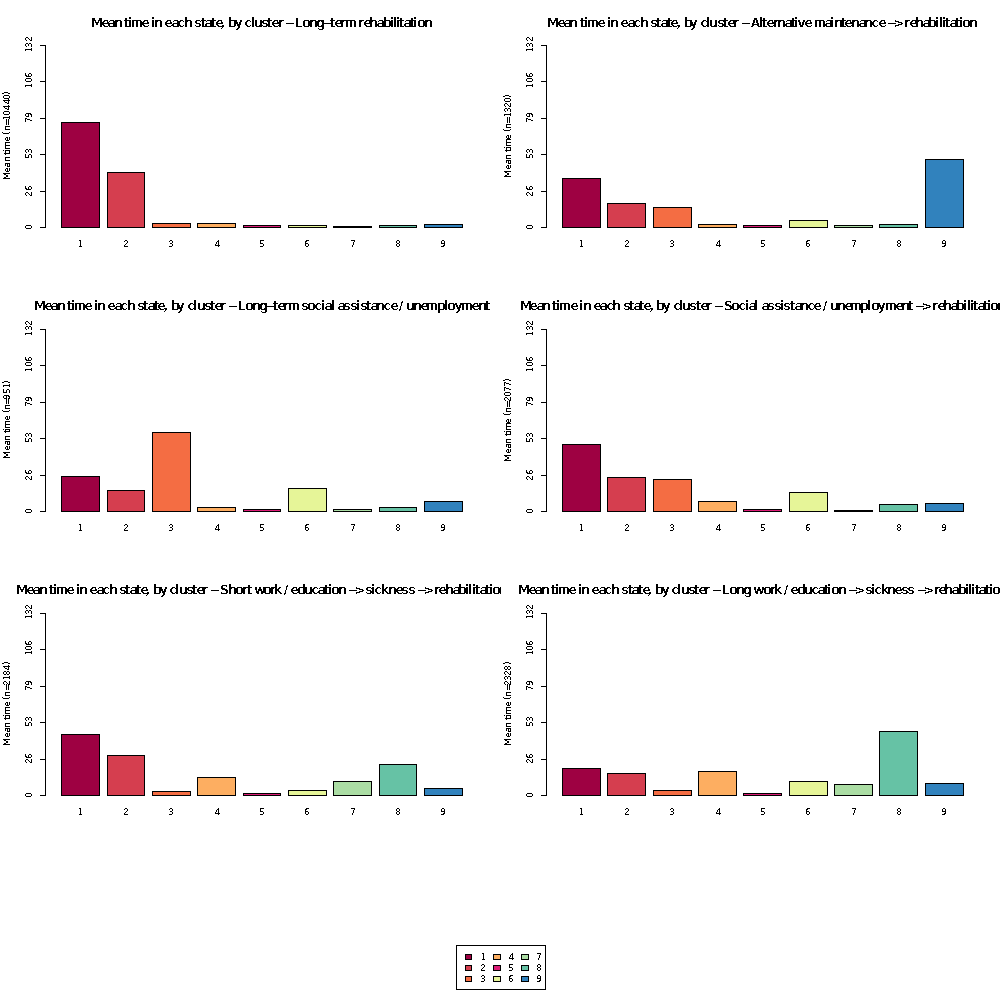


Figure A4. Mean time in each state for Cohort 1, by cluster


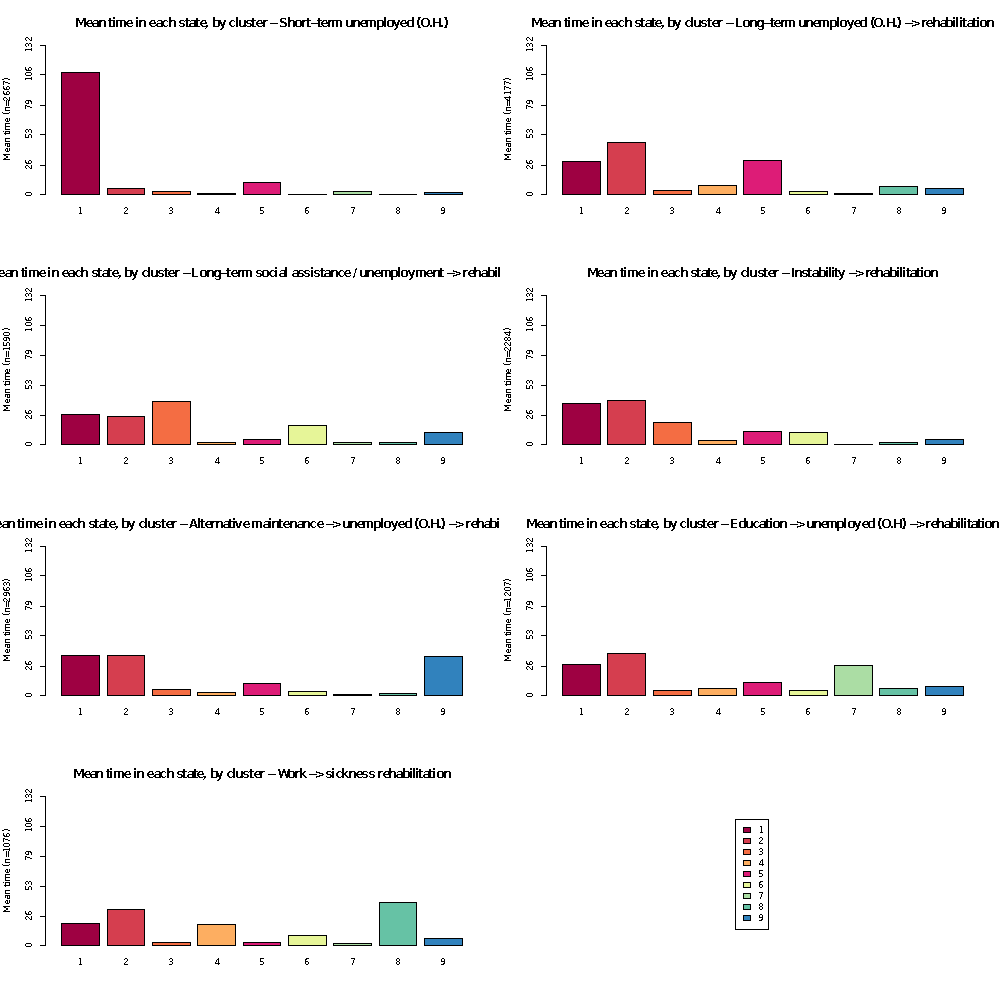

Supplement: Multimedia component 1 [file mmc1.docx]
